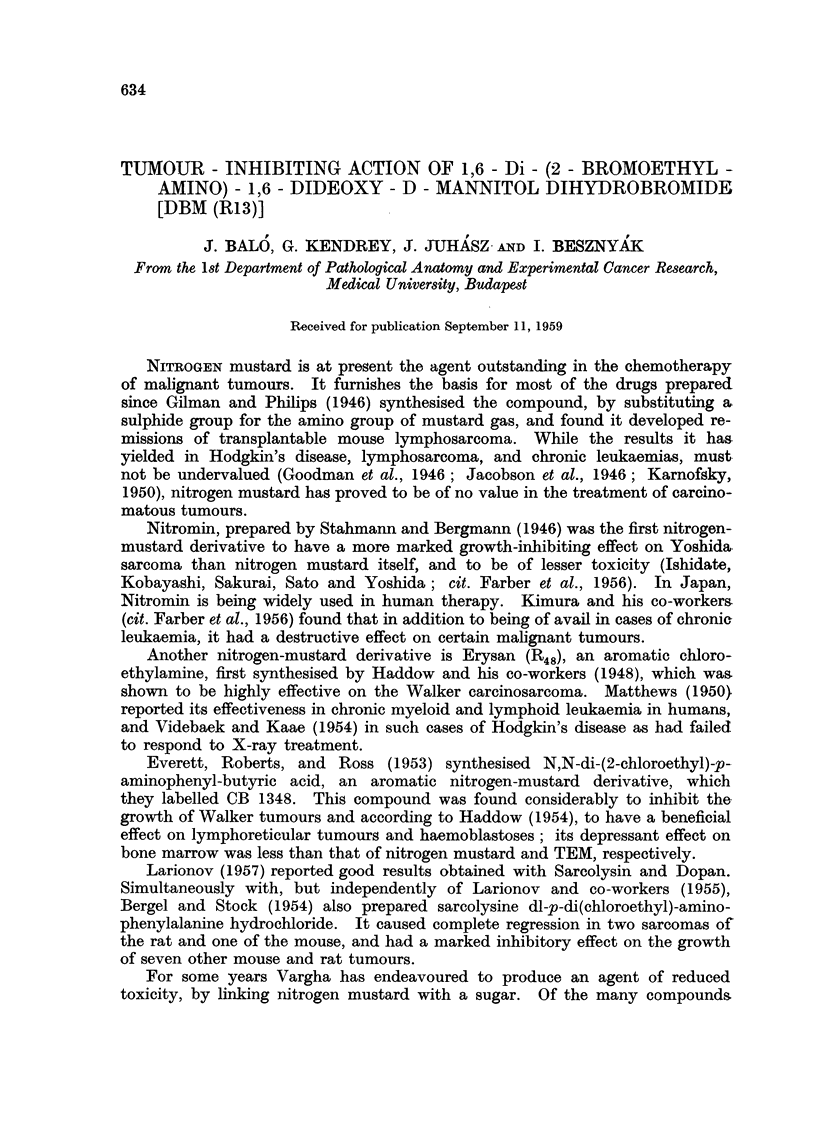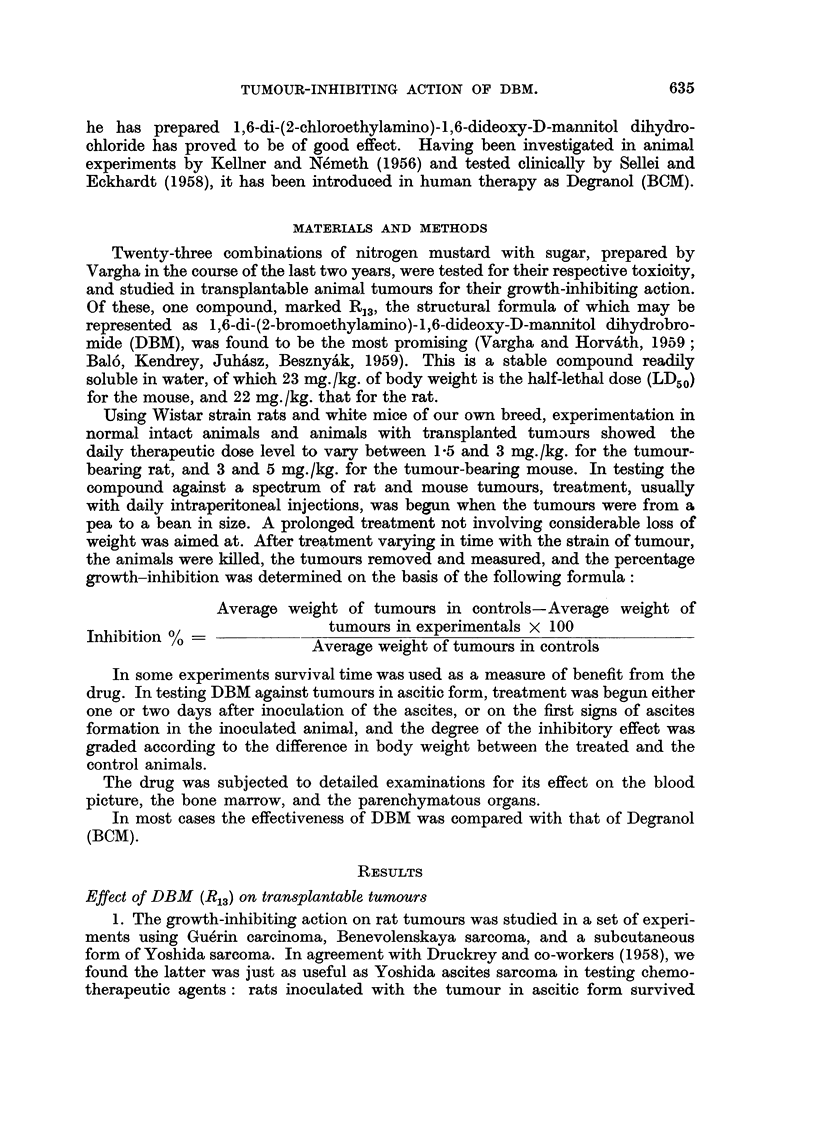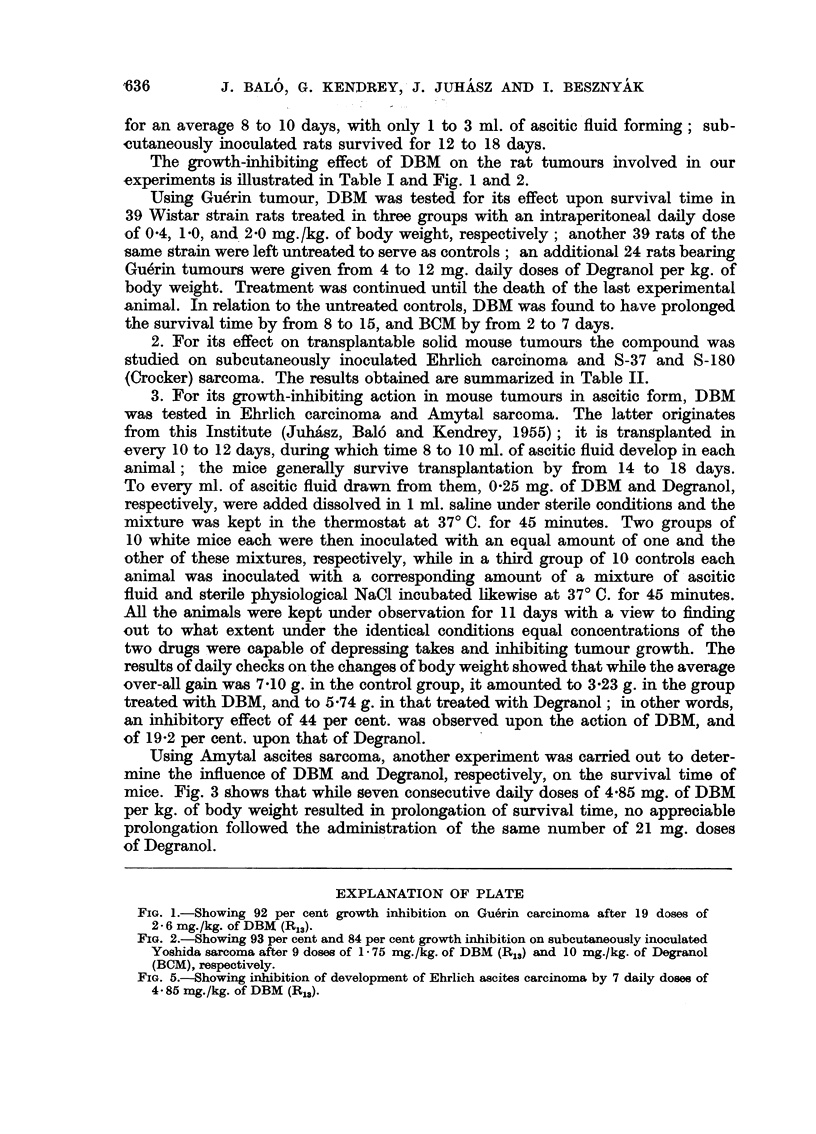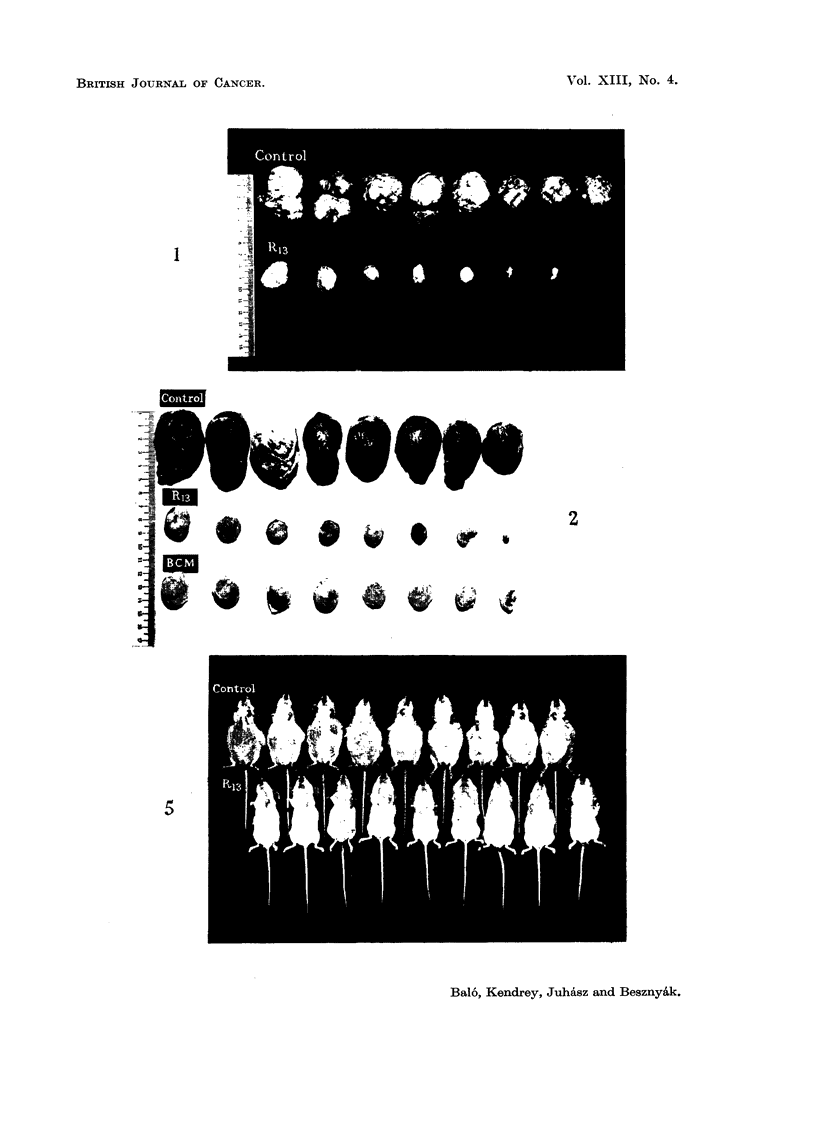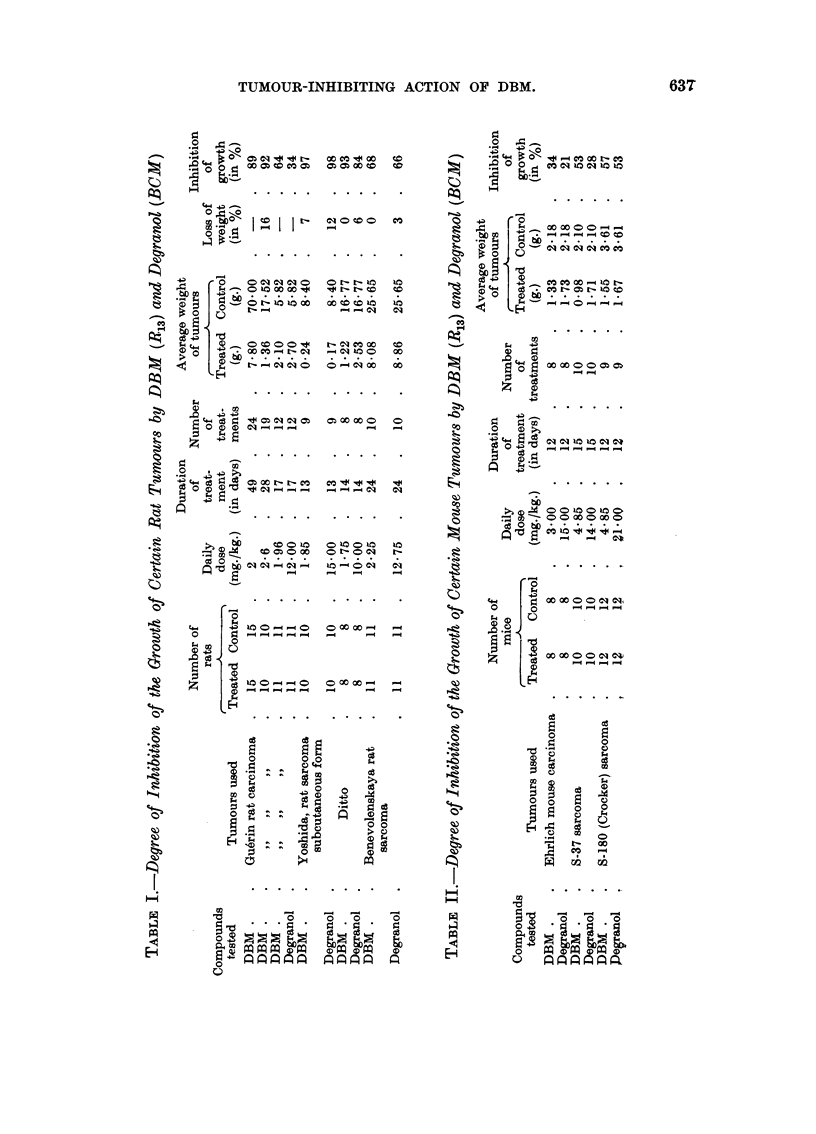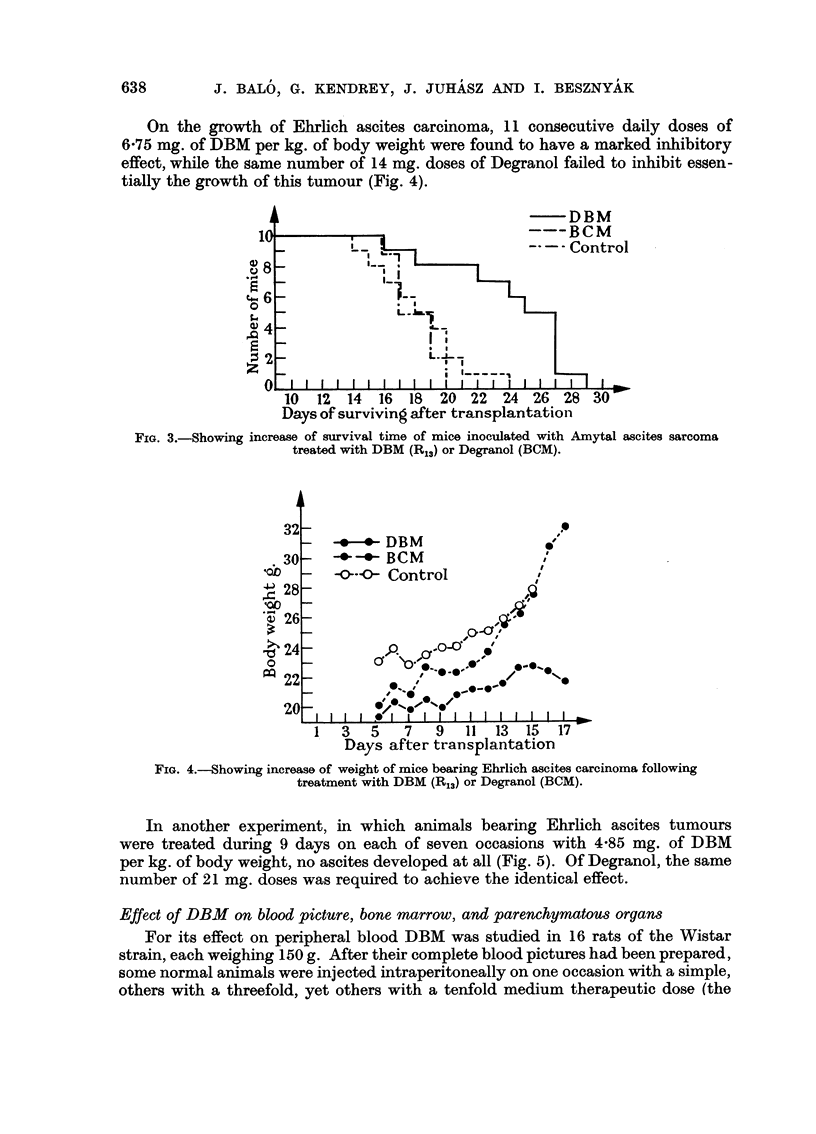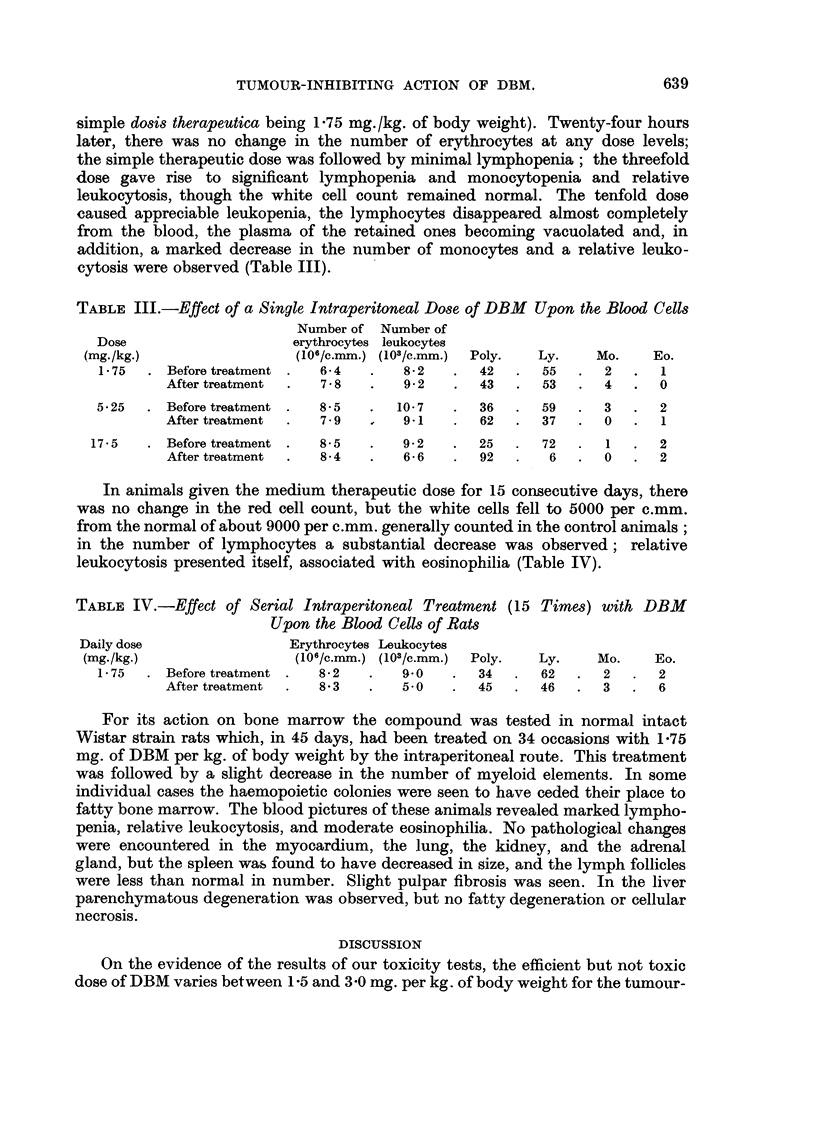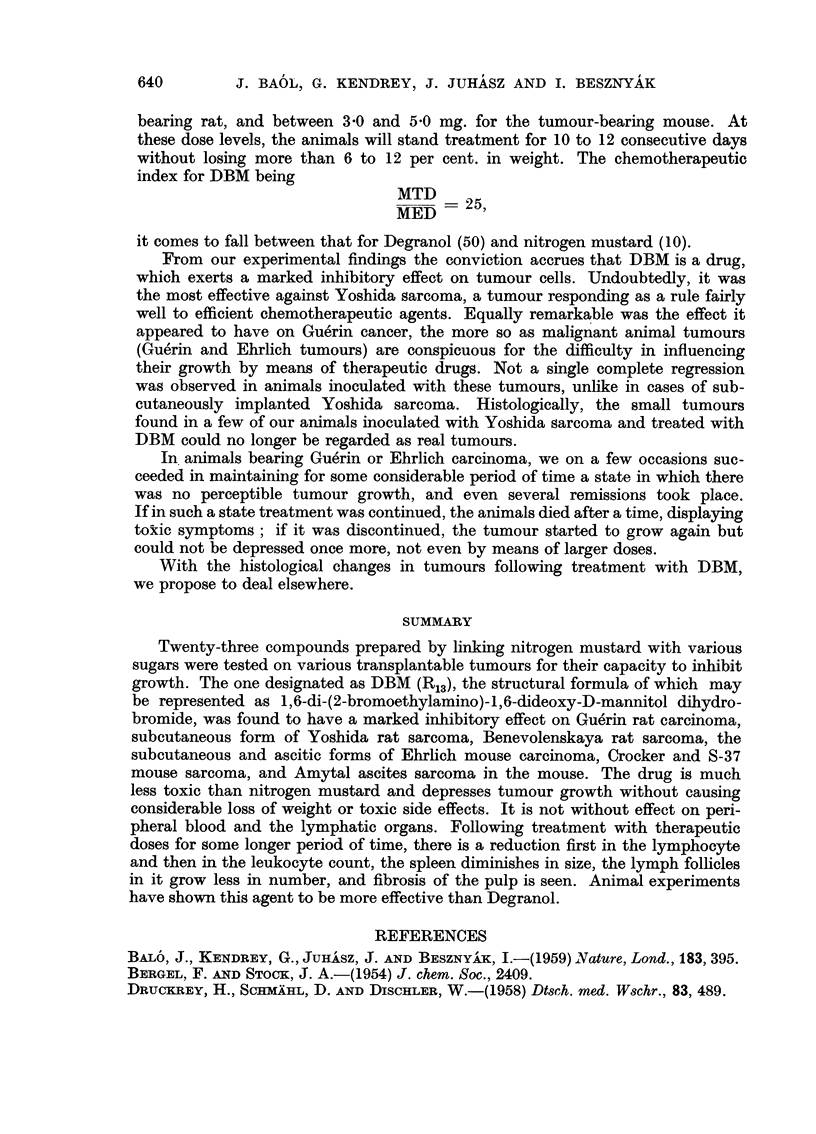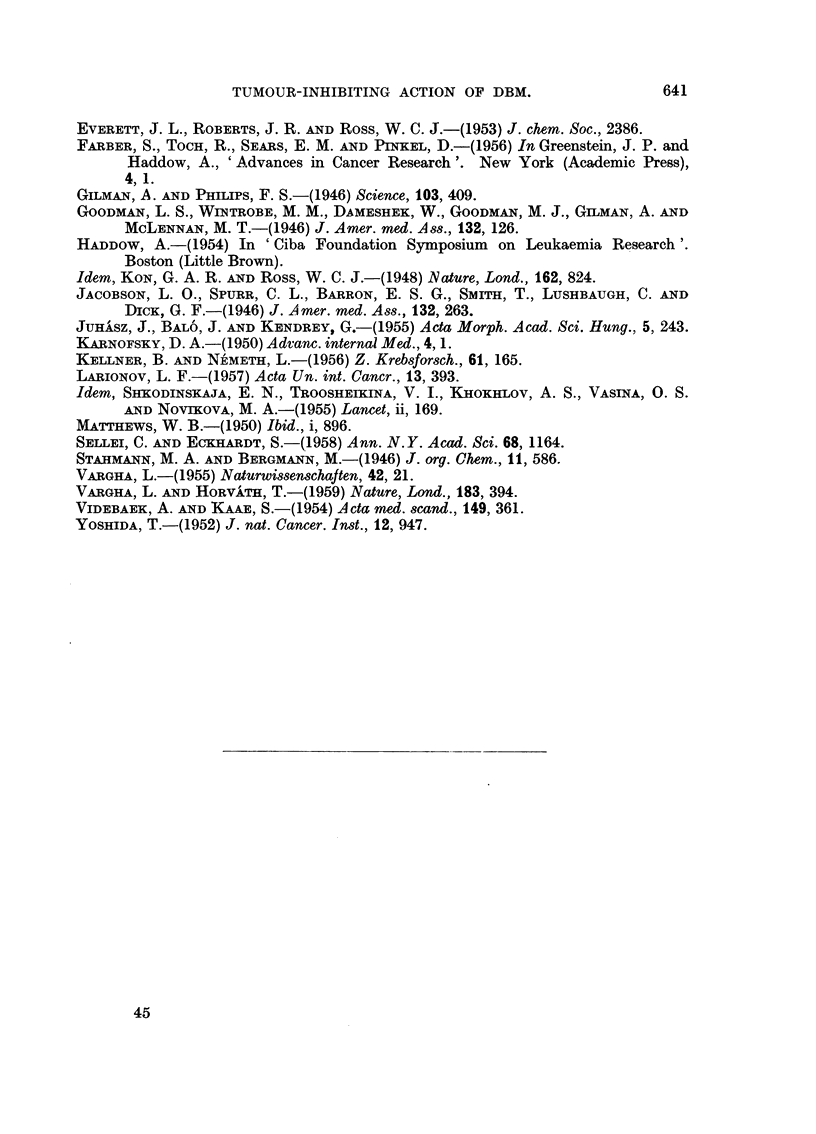# Tumour-inhibiting Action of 1,6-Di-(2-Bromoethylamino)-1,6-Dideoxy-D-Mannitol Dihydrobromide [DBM (R13)]

**DOI:** 10.1038/bjc.1959.69

**Published:** 1959-12

**Authors:** J. Baló, G. Kendrey, J. Juhász, I. Besznyák

## Abstract

**Images:**


### TUMOUR - INHIBITING ACTION OF 1,6 - Di - (2 - BROMOETHYL - AMINO) - 1,6 - DIDEOXY - D - MANNITOL DIHYDROBROMIDE [DBM (R13)]

J. BALÓ, G. KENDREY, J. JUHÁSZ AND I. BESZNYÁK

*From the 1st Department of Pathological Anatomy and Experimental Cancer Research, Medical University, Budapest*

Received for publication September 11, 1959

NITROGEN mustard is at present the agent outstanding in the chemotherapy of malignant tumours. It furnishes the basis for most of the drugs prepared since Gilman and Philips (1946) synthesised the compound, by substituting a sulphide group for the amino group of mustard gas, and found it developed remissions of transplantable mouse lymphosarcoma. While the results it has yielded in Hodgkin's disease, lymphosarcoma, and chronic leukaemias, must not be undervalued (Goodman *et al.*, 1946 ; Jacobson *et al.*, 1946 ; Karnofsky, 1950), nitrogen mustard has proved to be of no value in the treatment of carcinomatous tumours.

Nitromin, prepared by Stahmann and Bergmann (1946) was the first nitrogen-mustard derivative to have a more marked growth-inhibiting effect on Yoshida sarcoma than nitrogen mustard itself, and to be of lesser toxicity (Ishidate, Kobayashi, Sakurai, Sato and Yoshida ; *cit.* Farber *et al.*, 1956). In Japan, Nitromin is being widely used in human therapy. Kimura and his co-workers (*cit.* Farber *et al.*, 1956) found that in addition to being of avail in cases of chronic leukaemia, it had a destructive effect on certain malignant tumours.

Another nitrogen-mustard derivative is Erysan ($R_{48}$), an aromatic chloroethylamine, first synthesised by Haddow and his co-workers (1948), which was shown to be highly effective on the Walker carcinosarcoma. Matthews (1950) reported its effectiveness in chronic myeloid and lymphoid leukaemia in humans, and Videbaek and Kaae (1954) in such cases of Hodgkin's disease as had failed to respond to X-ray treatment.

Everett, Roberts, and Ross (1953) synthesised N,N-di-(2-chloroethyl)-*p*-aminophenyl-butyric acid, an aromatic nitrogen-mustard derivative, which they labelled CB 1348. This compound was found considerably to inhibit the growth of Walker tumours and according to Haddow (1954), to have a beneficial effect on lymphoreticular tumours and haemoblastoses ; its depressant effect on bone marrow was less than that of nitrogen mustard and TEM, respectively.

Larionov (1957) reported good results obtained with Sarcolysin and Dopan. Simultaneously with, but independently of Larionov and co-workers (1955), Bergel and Stock (1954) also prepared sarcolysine dl-*p*-di(chloroethyl)-amino-phenylalanine hydrochloride. It caused complete regression in two sarcomas of the rat and one of the mouse, and had a marked inhibitory effect on the growth of seven other mouse and rat tumours.

For some years Vargha has endeavoured to produce an agent of reduced toxicity, by linking nitrogen mustard with a sugar. Of the many compounds he has prepared 1,6-di-(2-chloroethylamino)-1,6-dideoxy-D-mannitol dihydrochloride has proved to be of good effect. Having been investigated in animal experiments by Kellner and Németh (1956) and tested clinically by Sellei and Eckhardt (1958), it has been introduced in human therapy as Degranol (BCM).

### MATERIALS AND METHODS

Twenty-three combinations of nitrogen mustard with sugar, prepared by Vargha in the course of the last two years, were tested for their respective toxicity, and studied in transplantable animal tumours for their growth-inhibiting action. Of these, one compound, marked $R_{13}$, the structural formula of which may be represented as 1,6-di-(2-bromoethylamino)-1,6-dideoxy-D-mannitol dihydrobromide (DBM), was found to be the most promising (Vargha and Horváth, 1959; Baló, Kendrey, Juhász, Besznyák, 1959). This is a stable compound readily soluble in water, of which 23 mg./kg. of body weight is the half-lethal dose ($LD_{50}$) for the mouse, and 22 mg./kg. that for the rat.

Using Wistar strain rats and white mice of our own breed, experimentation in normal intact animals and animals with transplanted tumours showed the daily therapeutic dose level to vary between 1·5 and 3 mg./kg. for the tumour-bearing rat, and 3 and 5 mg./kg. for the tumour-bearing mouse. In testing the compound against a spectrum of rat and mouse tumours, treatment, usually with daily intraperitoneal injections, was begun when the tumours were from a pea to a bean in size. A prolonged treatment not involving considerable loss of weight was aimed at. After treatment varying in time with the strain of tumour, the animals were killed, the tumours removed and measured, and the percentage growth–inhibition was determined on the basis of the following formula:

$$\text{Inhibition } \% = \frac{\text{Average weight of tumours in controls} - \text{Average weight of tumours in experimentals} \times 100}{\text{Average weight of tumours in controls}}$$

In some experiments survival time was used as a measure of benefit from the drug. In testing DBM against tumours in ascitic form, treatment was begun either one or two days after inoculation of the ascites, or on the first signs of ascites formation in the inoculated animal, and the degree of the inhibitory effect was graded according to the difference in body weight between the treated and the control animals.

The drug was subjected to detailed examinations for its effect on the blood picture, the bone marrow, and the parenchymatous organs.

In most cases the effectiveness of DBM was compared with that of Degranol (BCM).

### RESULTS

### *Effect of DBM ($R_{13}$) on transplantable tumours*

1. The growth-inhibiting action on rat tumours was studied in a set of experiments using Guérin carcinoma, Benevolenskaya sarcoma, and a subcutaneous form of Yoshida sarcoma. In agreement with Druckrey and co-workers (1958), we found the latter was just as useful as Yoshida ascites sarcoma in testing chemotherapeutic agents: rats inoculated with the tumour in ascitic form survived for an average 8 to 10 days, with only 1 to 3 ml. of ascitic fluid forming ; subcutaneously inoculated rats survived for 12 to 18 days.

The growth-inhibiting effect of DBM on the rat tumours involved in our experiments is illustrated in Table I and Fig. 1 and 2.

Using Guérin tumour, DBM was tested for its effect upon survival time in 39 Wistar strain rats treated in three groups with an intraperitoneal daily dose of 0·4, 1·0, and 2·0 mg./kg. of body weight, respectively ; another 39 rats of the same strain were left untreated to serve as controls ; an additional 24 rats bearing Guérin tumours were given from 4 to 12 mg. daily doses of Degranol per kg. of body weight. Treatment was continued until the death of the last experimental animal. In relation to the untreated controls, DBM was found to have prolonged the survival time by from 8 to 15, and BCM by from 2 to 7 days.

2. For its effect on transplantable solid mouse tumours the compound was studied on subcutaneously inoculated Ehrlich carcinoma and S-37 and S-180 (Crocker) sarcoma. The results obtained are summarized in Table II.

3. For its growth-inhibiting action in mouse tumours in ascitic form, DBM was tested in Ehrlich carcinoma and Amytal sarcoma. The latter originates from this Institute (Juhász, Baló and Kendrey, 1955) ; it is transplanted in every 10 to 12 days, during which time 8 to 10 ml. of ascitic fluid develop in each animal ; the mice generally survive transplantation by from 14 to 18 days. To every ml. of ascitic fluid drawn from them, 0·25 mg. of DBM and Degranol, respectively, were added dissolved in 1 ml. saline under sterile conditions and the mixture was kept in the thermostat at 37° C. for 45 minutes. Two groups of 10 white mice each were then inoculated with an equal amount of one and the other of these mixtures, respectively, while in a third group of 10 controls each animal was inoculated with a corresponding amount of a mixture of ascitic fluid and sterile physiological NaCl incubated likewise at 37° C. for 45 minutes. All the animals were kept under observation for 11 days with a view to finding out to what extent under the identical conditions equal concentrations of the two drugs were capable of depressing takes and inhibiting tumour growth. The results of daily checks on the changes of body weight showed that while the average over-all gain was 7·10 g. in the control group, it amounted to 3·23 g. in the group treated with DBM, and to 5·74 g. in that treated with Degranol ; in other words, an inhibitory effect of 44 per cent. was observed upon the action of DBM, and of 19·2 per cent. upon that of Degranol.

Using Amytal ascites sarcoma, another experiment was carried out to determine the influence of DBM and Degranol, respectively, on the survival time of mice. Fig. 3 shows that while seven consecutive daily doses of 4·85 mg. of DBM per kg. of body weight resulted in prolongation of survival time, no appreciable prolongation followed the administration of the same number of 21 mg. doses of Degranol.

---

### EXPLANATION OF PLATE

FIG. 1.—Showing 92 per cent growth inhibition on Guérin carcinoma after 19 doses of 2·6 mg./kg. of DBM ($R_{13}$).

FIG. 2.—Showing 93 per cent and 84 per cent growth inhibition on subcutaneously inoculated Yoshida sarcoma after 9 doses of 1·75 mg./kg. of DBM ($R_{13}$) and 10 mg./kg. of Degranol (BCM), respectively.

FIG. 5.—Showing inhibition of development of Ehrlich ascites carcinoma by 7 daily doses of 4·85 mg./kg. of DBM ($R_{13}$).

1

2

5

Baló, Kendrey, Juhász and Besznyák.

TABLE I.—*Degree of Inhibition of the Growth of Certain Rat Tumours by DBM ($R_{13}$) and Degranol (BCM)*

| Compounds tested | Tumours used | Number of rats: Treated | Number of rats: Control | Daily dose (mg./kg.) | Duration of treatment (in days) | Number of treatments | Average weight of tumours: Treated (g.) | Average weight of tumours: Control (g.) | Loss of weight (in %) | Inhibition of growth (in %) |
|---|---|---|---|---|---|---|---|---|---|---|
| DBM | Guérin rat carcinoma | 15 | 15 | 2 | 49 | 24 | 7·80 | 70·00 | — | 89 |
| DBM | ,, ,, ,, | 10 | 10 | 2·6 | 28 | 19 | 1·36 | 17·52 | 16 | 92 |
| DBM | ,, ,, ,, | 11 | 11 | 1·96 | 17 | 12 | 2·10 | 5·82 | — | 64 |
| Degranol | | 11 | 11 | 12·00 | 17 | 12 | 2·70 | 5·82 | — | 34 |
| DBM | Yoshida, rat sarcoma subcutaneous form | 10 | 10 | 1·85 | 13 | 9 | 0·24 | 8·40 | 7 | 97 |
| Degranol | | 10 | 10 | 15·00 | 13 | 9 | 0·17 | 8·40 | 12 | 98 |
| DBM | Ditto | 8 | 8 | 1·75 | 14 | 8 | 1·22 | 16·77 | 0 | 93 |
| Degranol | | 8 | 8 | 10·00 | 14 | 8 | 2·53 | 16·77 | 6 | 84 |
| DBM | Benevolenskaya rat sarcoma | 11 | 11 | 2·25 | 24 | 10 | 8·08 | 25·65 | 0 | 68 |
| Degranol | | 11 | 11 | 12·75 | 24 | 10 | 8·86 | 25·65 | 3 | 66 |

TABLE II.—*Degree of Inhibition of the Growth of Certain Mouse Tumours by DBM ($R_{13}$) and Degranol (BCM)*

| Compounds tested | Tumours used | Number of mice: Treated | Number of mice: Control | Daily dose (mg./kg.) | Duration of treatment (in days) | Number of treatments | Average weight of tumours: Treated (g.) | Average weight of tumours: Control (g.) | Inhibition of growth (in %) |
|---|---|---|---|---|---|---|---|---|---|
| DBM | Ehrlich mouse carcinoma | 8 | 8 | 3·00 | 12 | 8 | 1·33 | 2·18 | 34 |
| Degranol | | 8 | 8 | 15·00 | 12 | 8 | 1·73 | 2·18 | 21 |
| DBM | S-37 sarcoma | 10 | 10 | 4·85 | 15 | 10 | 0·98 | 2·10 | 53 |
| Degranol | | 10 | 10 | 14·00 | 15 | 10 | 1·71 | 2·10 | 28 |
| DBM | S-180 (Crocker) sarcoma | 12 | 12 | 4·85 | 12 | 9 | 1·55 | 3·61 | 57 |
| Degranol | | 12 | 12 | 21·00 | 12 | 9 | 1·67 | 3·61 | 53 |

On the growth of Ehrlich ascites carcinoma, 11 consecutive daily doses of 6·75 mg. of DBM per kg. of body weight were found to have a marked inhibitory effect, while the same number of 14 mg. doses of Degranol failed to inhibit essentially the growth of this tumour (Fig. 4).

FIG. 3.—Showing increase of survival time of mice inoculated with Amytal ascites sarcoma treated with DBM ($R_{13}$) or Degranol (BCM).

FIG. 4.—Showing increase of weight of mice bearing Ehrlich ascites carcinoma following treatment with DBM ($R_{13}$) or Degranol (BCM).

In another experiment, in which animals bearing Ehrlich ascites tumours were treated during 9 days on each of seven occasions with 4·85 mg. of DBM per kg. of body weight, no ascites developed at all (Fig. 5). Of Degranol, the same number of 21 mg. doses was required to achieve the identical effect.

*Effect of DBM on blood picture, bone marrow, and parenchymatous organs*

For its effect on peripheral blood DBM was studied in 16 rats of the Wistar strain, each weighing 150 g. After their complete blood pictures had been prepared, some normal animals were injected intraperitoneally on one occasion with a simple, others with a threefold, yet others with a tenfold medium therapeutic dose (the simple *dosis therapeutica* being 1·75 mg./kg. of body weight). Twenty-four hours later, there was no change in the number of erythrocytes at any dose levels; the simple therapeutic dose was followed by minimal lymphopenia ; the threefold dose gave rise to significant lymphopenia and monocytopenia and relative leukocytosis, though the white cell count remained normal. The tenfold dose caused appreciable leukopenia, the lymphocytes disappeared almost completely from the blood, the plasma of the retained ones becoming vacuolated and, in addition, a marked decrease in the number of monocytes and a relative leukocytosis were observed (Table III).

TABLE III.—*Effect of a Single Intraperitoneal Dose of DBM Upon the Blood Cells*

| Dose (mg./kg.) | | Number of erythrocytes ($10^6$/c.mm.) | Number of leukocytes ($10^3$/c.mm.) | Poly. | Ly. | Mo. | Eo. |
|---|---|---|---|---|---|---|---|
| 1·75 | Before treatment | 6·4 | 8·2 | 42 | 55 | 2 | 1 |
| | After treatment | 7·8 | 9·2 | 43 | 53 | 4 | 0 |
| 5·25 | Before treatment | 8·5 | 10·7 | 36 | 59 | 3 | 2 |
| | After treatment | 7·9 | 9·1 | 62 | 37 | 0 | 1 |
| 17·5 | Before treatment | 8·5 | 9·2 | 25 | 72 | 1 | 2 |
| | After treatment | 8·4 | 6·6 | 92 | 6 | 0 | 2 |

In animals given the medium therapeutic dose for 15 consecutive days, there was no change in the red cell count, but the white cells fell to 5000 per c.mm. from the normal of about 9000 per c.mm. generally counted in the control animals ; in the number of lymphocytes a substantial decrease was observed ; relative leukocytosis presented itself, associated with eosinophilia (Table IV).

TABLE IV.—*Effect of Serial Intraperitoneal Treatment* (15 *Times*) *with DBM Upon the Blood Cells of Rats*

| Daily dose (mg./kg.) | | Erythrocytes ($10^6$/c.mm.) | Leukocytes ($10^3$/c.mm.) | Poly. | Ly. | Mo. | Eo. |
|---|---|---|---|---|---|---|---|
| 1·75 | Before treatment | 8·2 | 9·0 | 34 | 62 | 2 | 2 |
| | After treatment | 8·3 | 5·0 | 45 | 46 | 3 | 6 |

For its action on bone marrow the compound was tested in normal intact Wistar strain rats which, in 45 days, had been treated on 34 occasions with 1·75 mg. of DBM per kg. of body weight by the intraperitoneal route. This treatment was followed by a slight decrease in the number of myeloid elements. In some individual cases the haemopoietic colonies were seen to have ceded their place to fatty bone marrow. The blood pictures of these animals revealed marked lymphopenia, relative leukocytosis, and moderate eosinophilia. No pathological changes were encountered in the myocardium, the lung, the kidney, and the adrenal gland, but the spleen was found to have decreased in size, and the lymph follicles were less than normal in number. Slight pulpar fibrosis was seen. In the liver parenchymatous degeneration was observed, but no fatty degeneration or cellular necrosis.

### DISCUSSION

On the evidence of the results of our toxicity tests, the efficient but not toxic dose of DBM varies between 1·5 and 3·0 mg. per kg. of body weight for the tumourbearing rat, and between 3·0 and 5·0 mg. for the tumour-bearing mouse. At these dose levels, the animals will stand treatment for 10 to 12 consecutive days without losing more than 6 to 12 per cent. in weight. The chemotherapeutic index for DBM being

$$\frac{\text{MTD}}{\text{MED}} = 25,$$

it comes to fall between that for Degranol (50) and nitrogen mustard (10).

From our experimental findings the conviction accrues that DBM is a drug, which exerts a marked inhibitory effect on tumour cells. Undoubtedly, it was the most effective against Yoshida sarcoma, a tumour responding as a rule fairly well to efficient chemotherapeutic agents. Equally remarkable was the effect it appeared to have on Guérin cancer, the more so as malignant animal tumours (Guérin and Ehrlich tumours) are conspicuous for the difficulty in influencing their growth by means of therapeutic drugs. Not a single complete regression was observed in animals inoculated with these tumours, unlike in cases of subcutaneously implanted Yoshida sarcoma. Histologically, the small tumours found in a few of our animals inoculated with Yoshida sarcoma and treated with DBM could no longer be regarded as real tumours.

In animals bearing Guérin or Ehrlich carcinoma, we on a few occasions succeeded in maintaining for some considerable period of time a state in which there was no perceptible tumour growth, and even several remissions took place. If in such a state treatment was continued, the animals died after a time, displaying toxic symptoms ; if it was discontinued, the tumour started to grow again but could not be depressed once more, not even by means of larger doses.

With the histological changes in tumours following treatment with DBM, we propose to deal elsewhere.

### SUMMARY

Twenty-three compounds prepared by linking nitrogen mustard with various sugars were tested on various transplantable tumours for their capacity to inhibit growth. The one designated as DBM ($R_{13}$), the structural formula of which may be represented as 1,6-di-(2-bromoethylamino)-1,6-dideoxy-D-mannitol dihydrobromide, was found to have a marked inhibitory effect on Guérin rat carcinoma, subcutaneous form of Yoshida rat sarcoma, Benevolenskaya rat sarcoma, the subcutaneous and ascitic forms of Ehrlich mouse carcinoma, Crocker and S-37 mouse sarcoma, and Amytal ascites sarcoma in the mouse. The drug is much less toxic than nitrogen mustard and depresses tumour growth without causing considerable loss of weight or toxic side effects. It is not without effect on peripheral blood and the lymphatic organs. Following treatment with therapeutic doses for some longer period of time, there is a reduction first in the lymphocyte and then in the leukocyte count, the spleen diminishes in size, the lymph follicles in it grow less in number, and fibrosis of the pulp is seen. Animal experiments have shown this agent to be more effective than Degranol.

### REFERENCES

BALÓ, J., KENDREY, G., JUHÁSZ, J. AND BESZNYÁK, I.—(1959) *Nature, Lond.*, **183**, 395.
BERGEL, F. AND STOCK, J. A.—(1954) *J. chem. Soc.*, 2409.
DRUCKREY, H., SCHMÄHL, D. AND DISCHLER, W.—(1958) *Dtsch. med. Wschr.*, **83**, 489.

EVERETT, J. L., ROBERTS, J. R. AND ROSS, W. C. J.—(1953) *J. chem. Soc.*, 2386.
FARBER, S., TOCH, R., SEARS, E. M. AND PINKEL, D.—(1956) *In* Greenstein, J. P. and Haddow, A., 'Advances in Cancer Research'. New York (Academic Press), **4**, 1.
GILMAN, A. AND PHILIPS, F. S.—(1946) *Science*, **103**, 409.
GOODMAN, L. S., WINTROBE, M. M., DAMESHEK, W., GOODMAN, M. J., GILMAN, A. AND McLENNAN, M. T.—(1946) *J. Amer. med. Ass.*, **132**, 126.
HADDOW, A.—(1954) In 'Ciba Foundation Symposium on Leukaemia Research'. Boston (Little Brown).
*Idem*, KON, G. A. R. AND ROSS, W. C. J.—(1948) *Nature, Lond.*, **162**, 824.
JACOBSON, L. O., SPURR, C. L., BARRON, E. S. G., SMITH, T., LUSHBAUGH, C. AND DICK, G. F.—(1946) *J. Amer. med. Ass.*, **132**, 263.
JUHÁSZ, J., BALÓ, J. AND KENDREY, G.—(1955) *Acta Morph. Acad. Sci. Hung.*, **5**, 243.
KARNOFSKY, D. A.—(1950) *Advanc. internal Med.*, **4**, 1.
KELLNER, B. AND NÉMETH, L.—(1956) *Z. Krebsforsch.*, **61**, 165.
LARIONOV, L. F.—(1957) *Acta Un. int. Cancr.*, **13**, 393.
*Idem*, SHKODINSKAJA, E. N., TROOSHEIKINA, V. I., KHOKHLOV, A. S., VASINA, O. S. AND NOVIKOVA, M. A.—(1955) *Lancet*, ii, 169.
MATTHEWS, W. B.—(1950) *Ibid.*, i, 896.
SELLEI, C. AND ECKHARDT, S.—(1958) *Ann. N.Y. Acad. Sci.* **68**, 1164.
STAHMANN, M. A. AND BERGMANN, M.—(1946) *J. org. Chem.*, **11**, 586.
VARGHA, L.—(1955) *Naturwissenschaften*, **42**, 21.
VARGHA, L. AND HORVÁTH, T.—(1959) *Nature, Lond.*, **183**, 394.
VIDEBAEK, A. AND KAAE, S.—(1954) *Acta med. scand.*, **149**, 361.
YOSHIDA, T.—(1952) *J. nat. Cancer. Inst.*, **12**, 947.